# Exercise Performance Upregulatory Effect of R-α-Lipoic Acid with γ-Cyclodextrin

**DOI:** 10.3390/nu14010021

**Published:** 2021-12-22

**Authors:** Yuki Hashimoto, Katsuhiko Yoshizawa, Yuka Kaido, Akiko Takenouchi, Keiji Terao, Hiroyuki Yasui, Yutaka Yoshikawa

**Affiliations:** 1 Department of Health Sports Nutrition, Faculty of Health and Welfare, Kobe Women’s University, Hyogo 650-0046, Japan; y-hashimoto@yg.kobe-wu.ac.jp (Y.H.); k3318017@suma.kobe-wu.ac.jp (Y.K.); 2 Department of Innovative Food Sciences, School of Food Sciences and Nutrition, Mukogawa Women’s University, Hyogo 663-8183, Japan; yoshizak@mukogawa-u.ac.jp (K.Y.); takeko@mukogawa-u.ac.jp (A.T.); 3 CycloChem Company Limited, Hyogo 650-0047, Japan; keiji.terao@cyclochem.com; 4 Department of Analytical and Bioinorganic Chemistry, Kyoto Pharmaceutical University, 5 Misasagi, Nakautityo, Yamashina-ku, Kyoto 607-8414, Japan; yasui@mb.kyoto-phu.ac.jp

**Keywords:** α-lipoic acid, γ-cyclodextrin complex, swimming exercise, oxidative stress

## Abstract

α-Lipoic acid (ALA) is a vitamin-like substance that is an indispensable supporting factor for a large number of enzymes. Due to its optical activity, ALA has optical isomers RALA and SALA. The major role of RALA is in energy metabolism. However, RALA cannot be used as a pharmaceutical or nutraceutical because it is sensitive to heat and acid conditions. Previous studies have shown that RALA complexed with γ-cyclodextrin (CD) has a higher antioxidant capacity than that of free RALA. The antioxidant enzyme system protects against intense exercise-induced oxidative damage and is related to the physical status of athletes. The aim of this study was to examine the effect of CD/RALA complex supplementation on antioxidant activity and performance during high-intensity exercise. Twenty-four male C3H/HeSlc mice were divided into four groups (*n* = 6): swimming+distilled water administration (C), swimming+CD/RALA supplementation (CD/RALA), swimming+RALA suplementation (RALA), and swimming+CD supplementation (CD). Blood ammonia elevation due to exercise stress was repressed by CD/RALA supplementation. The oxidative stress in the kidney increased after exercise and was reduced by CD/RALA supplementation. Our findings suggest that CD/RALA supplementation may be useful for improving the exercise performance in athletes.

## 1. Introduction

α-Lipoic acid (ALA) is a vitamin-like substance that is an indispensable supporting factor for a large number of enzymes [1]. It is present intracellularly in mitochondria and is widely distributed in living organisms [2,3]. It has been reported that ALA has antioxidant and anti-inflammatory effects, as well as preventive effects on lifestyle-related diseases such as cancer, arteriosclerosis, and diabetes mellitus [4,5,6]. It is currently used in various medicines and health-promoting foods [7,8,9].

**Figure 1:**
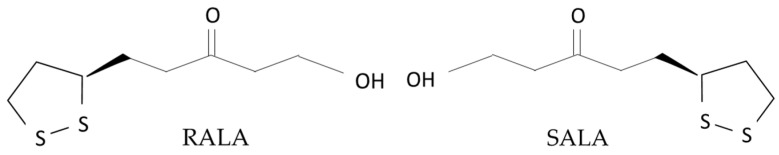
Chemical formulas of RALA and SALA.

ALA has optical isomers due to the presence of chiral carbons, which are of two types: R (RALA) and S (SALA) (Figure 1). RALA is a naturally occurring form that is considered to have high biological activity [10]. In contrast, SALA has been reported to aggravate diabetes and is considered to be a potential cause of health problems [11]. However, free RALA is extremely unstable and weak in heat and acid, so it is used as a racemic mixture containing equal proportions of RALA and SALA (DLALA) in supplements and other products [12].

**Figure 2:**
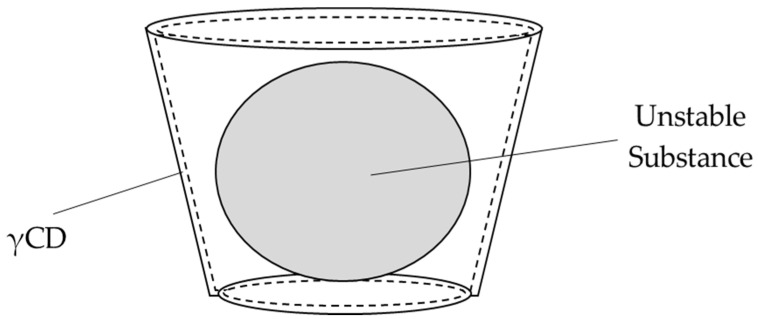
Illustration of γ-cyclodextrin inclusion.

Cyclodextrin (CD) is a cyclic oligosaccharide that is classified into α (6), β (7), and γ (8) according to the number of glucose chains [13]. γCD complexes have an interesting feature of stabilization of unstable substances by incorporating them into the hydrophobic cavity of γCD (Figure 2). It is possible to stabilize the unstable substance and increase its function by encapsulation [14]. It has been reported that the stability of RALA in gastric acid was improved by formation of γCD complex with RALA (γCD/RALA) [15].

Physical activity (PA) has been reported to protect against cardiovascular diseases (CVDs), cancer, metabolic syndrome, depression, anxiety, and cognitive/neurodegenerative disorders, collectively reducing all-cause mortality risk by approximately 30–40% [16,17,18,19,20,21]. Aerobic exercise training (AET) delays the onset of morbidity and enhances both health and lifespan [22]. Vigorous AET (for example, running) provides additional survival benefits of approximately 3–5 times the benefits of the recommended minimum PA (75–150 min/week), with up to 10-fold higher training volumes generally considered safe and well-tolerated [16,17,19,23].

However, there have been many reports on the adverse effects of high-impact exercise on the body; for example, increased oxidative stress due to increased production of reactive oxygen species (ROS), deficiency of minerals in the body, and associated symptoms such as iron deficiency anemia [24,25,26]. ROS, a general term for highly reactive compounds containing oxygen, plays an important role in biological defense [27]. However, an excessive ROS level has been reported to be involved in the onset of various diseases and aging [2]. In other words, a moderate ROS level is necessary for living organisms, but an excessive ROS level causes adverse effects in living organisms; as a result, compounds having an antioxidant effect are attracting attention. Thus, exercise performance can be improved by elimination of these negative effects.

ALA has potent antioxidant properties, and we believe that γCD enhances its effects. In fact, it has been reported that supplementation with a mixture of γCD/DLALA and γCD complexed with coenzyme Q10, rather than a mixture of DLALA and coenzyme Q10, prolonged swimming time during exercise with increased oxidative stress [28]. However, there are no reports on its effects during other types of exercise. In the present study, we investigated the effect of CDLA on the improvement of exercise performance.

## 2. Materials and Methods

### 2.1. Animals and Experimental Design

Male C3H/HeSlc mice aged 5 weeks were obtained from Japan SLC, Inc. (Osaka, Japan). All animals were housed in a temperature-controlled (22 ± 2 °C) environment under a 12 h light/dark cycle and had *ad libitum* access to food and water. The mice were fed a standard MF diet (Japan SLC, Inc.).

After 3 weeks, all mice practiced swimming for 10 min daily for a week in the Kyoto University Matsumoto swimming apparatus (Anitech Co., Ltd., Tokyo, Japan) at a water depth of 38 cm, water temperature of 32 °C, and flow rate of 10 L/min [28].

**Figure 3:**
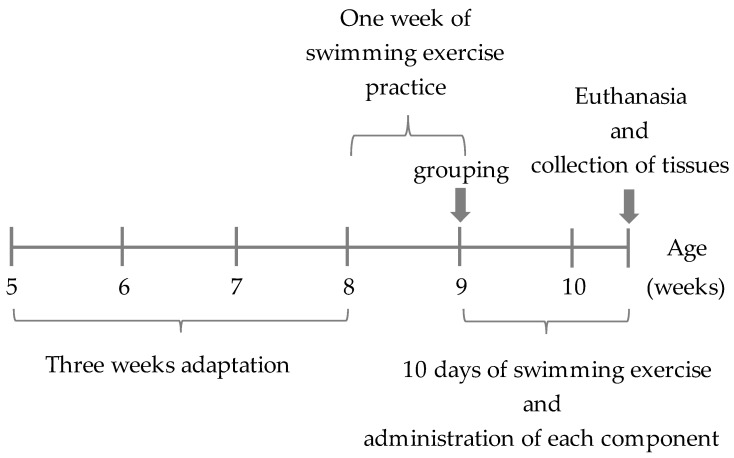
Experimental protocol.

After 4 weeks, the mice were divided into the following four groups based on oral administration of the samples before and after the swimming exercise: (1) distilled water administered (C), (2) CD/RALA administered (CD/RALA), (3) RALA administered (RALA), and (4) γCD-administered (CD). Samples (2) and (3) were prepared by dissolving them in methylcellulose. CD/RALA, RALA, and CD were provided by CycloChem Bio Co., Ltd. (Kobe, Japan). The doses of the various samples are listed in Table 1. Starting from the week 4, all mice exercised for 20 min daily for 10 days (Figure 3).

Body weight and food intake were measured daily. After 5 weeks, the mice were euthanized under isoflurane after measuring the swimming time to exhaustion. Blood was drawn from the abdominal aorta and transferred to heparin-coated vials. Plasma was prepared (1000 g, 10 min) and stored at −30 °C. The liver, kidney, spleen, lung, heart, quadriceps, and gastrocnemius were collected, weighted, and stored at −30 °C.

### 2.2. Blood Biochemical Analyses

Plasma ammonia (NH_3_) and creatinine phosphokinase (CPK) levels were measured using an automatic dry-chemistry analyzer (Fuji Dri-Chem 3500V; Fujifilm Medical, Tokyo, Japan).

### 2.3. Measurement of Artificial Superoxide Anion Production

The superoxide anion (O_2_^−^) is generated from the reaction between hypoxanthine and xanthine oxidase. For the hepatic and renal superoxide anion measurements, 2-methyl-6-*p*-methoxyphenylethnylimidazopyrazinone (MPEC) was used to induce oxidation. Xanthine oxidase and hypoxanthine were prepared in a phosphate buffer (0.1 M KH_2_PO_4_ buffer, pH 7.5). The reaction solution for the superoxide anion scavenging activity test consisted of 60 µL crude hepatic or renal enzyme solution, 10 µL 300 µM MPEC, 170 µL 0.1 M KH_2_PO_4_ buffer, 60 µL xanthine oxidase solution (0.1 U/mL), and 50 µL 3.6 mM hypoxanthine/KH_2_PO_4_. Crude hepatic or renal enzyme solution was obtained from sample homogenates prepared in phosphate-buffered saline (PBS) as follows: 0.1 g liver or kidney isolates was homogenized in 500 µL of 0.1 M PBS (pH 7.3), and the homogenized samples were collected and used as crude hepatic or renal enzyme solution. The reaction was initiated by adding hypoxanthine. Fifty microliters of the reaction solution was placed in each Röhren tube (5 mL; 75 mm × 12 mm; Sartedt, Nümbrecht, Germany) and MPEC light emission was measured using a luminometer (Lumat3 LB9508; Berthold Technologies, Bad Wildbad, Germany) [29].

### 2.4. Trace Element Analysis

The kidney sample (50 mg) was placed in 50 mL tall breaker and heated on a hotplate to 150 °C. Then, 2 mL of 60% (*v*/*v*) nitric acid (HNO_3_; Kanto Chemical, Tokyo, Japan) and 2 mL of 60% (*v*/*v*) perchloric acid (HClO_4_; Kishida Chemical, Osaka, Japan) were added. This process was repeated thrice. Next, 2 mL of 30% (*v*/*v*) H_2_O_2_ was added, and the sample was heated until digestion was complete. The liquid was evaporated, and the sample residues were cooled. Then, 9 mL of 5% (*v*/*v*) HNO_3_ was added, and the residues were dissolved for 3 h. The presence of iron (Fe) in the solution was identified and quantitated by inductively coupled plasma-mass spectrometry (ICP-MS; Agilent7700/Mass Hunter, Agilent Technologies, Santa Clara, CA, USA). Standard curve was plotted by preparing 1000 μg/mL (ppm) standard solution of Fe (Fujifilm Wako Pure Chemical Industries Ltd., Osaka, Japan) and diluting it in 5% (*v*/*v*) HNO_3_ to the final metal concentrations of 0, 1, 5, 10, 50, 100, and 500 ng/mL (ppb). For quality control, 1 ng/mL (ppb) of the reference internal standard, indium (In), was measured along with the sample [30].

### 2.5. Exercise Time to Exhaustion in Loading Swimming Test

At the age of 8 weeks, all mice were forced to swim, and the swimming time until fatigue was measured in all mice. A time point until fatigue was defined by the failure to rise to the surface of the water for more than 5 s to breathe. To avoid overstressing the mice, the maximum duration of the swimming exercise was set at one hour, and if it exceeded one hour, the mice were forcibly withdrawn from the pool [28,31,32].

### 2.6. Lactic Acid Measurement

At 5 weeks, blood was drawn from the caudal vein of the mice immediately before and after the completion of exercise training. Lactic acid level was determined using Lactate Pro2 (Arkray, Inc., Kyoto, Japan).

### 2.7. Histopathological and Immunohistochemical Examination of the Kidney

Kidneys were fixed overnight in methacarn (methanol: chloroform: acetic acid, 60:30:10 by volume) and then mounted in paraffin. Tissues were embedded in paraffin, sectioned at 4mm, and stained with hematoxylin and eosin (H&E). Sequential sections were degreased with xylene and a graded series of alcohol and then washed with phosphate-buffer saline (PBS) for 10 min. The carbonyl group was converted to DNP hydrazone by reaction with 1 mg/mL 2,4-dinitrophenylhydrazine (Cosmo Bio Co., Ltd., Tokyo, Japan) prepared in 2 N hydrochloric acid (HCl) for 30 min. Sections were washed with HCl and ethanol, blocked with 10% (*v*/*v*) normal goat serum, and subjected to microwave irradiation for 20 min, cooled at room temperature for 1 h, quenched with 1% H_2_O_2_/methanol solution, and washed three times with PBS. The cells were then reacted with the protein blocking serum to prevent non-specific reactions. The cells were then incubated overnight with rabbit anti-DNP (1:1000) (Nichirei Bioscience, Tokyo, Japan) at 4 °C. After removal of the primary antibody with PBS, the cells were incubated with Histofine^®^ Simple Stain MAX PO (Nichirei Bioscience) for 15 min. Sections were washed three times with PBS, stained with diaminobenzidine tablets (Nichirei Bioscience), and nuclear stained with hematoxylin solution after the reaction was stopped with distilled water [33].

### 2.8. Statistical Analysis

Data are expressed as mean ± SD. Means and standard deviations were calculated using Excel statistics (2012 version), and rejection tests were performed. The difference between the means of each sample was tested for significance by one-way analysis of variance, followed by multiple comparisons (Dunnett) with group C as control. Statistical significance was set at *p* ≤ 0.05. 

## 3. Results

### 3.1. General Characteristics

There were no significant differences in body weight and weight of various organs between the groups (Table 2). 

### 3.2. Blood Biochemical Analyses

**Figure 4:**
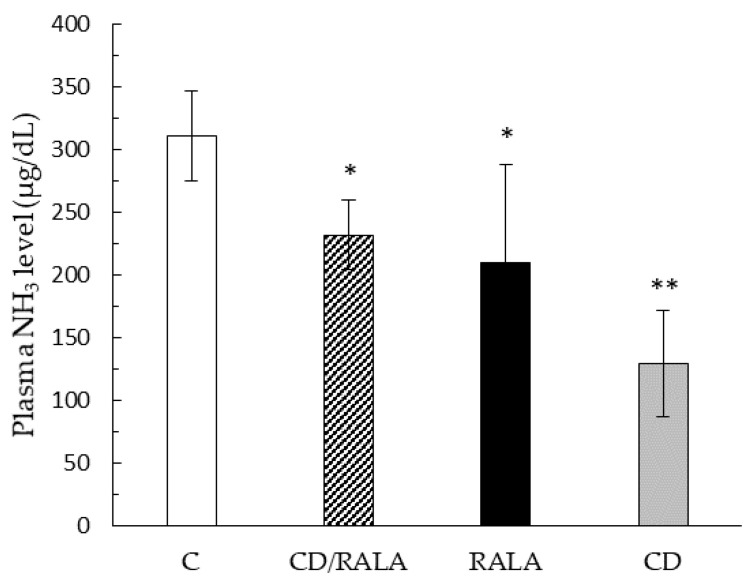
Blood ammonia concentration. Values are expressed as the mean SD for six mice. C, control group; CD/RALA, R-α-Lipoic acid γ-cyclodextrin complex group; RALA, R-α-Lipoc acid group; CD, γ-cyclodextrin group. ** *p* < 0.01 vs. C. * *p* < 0.05 vs. C.

Blood NH_3_ concentration was significantly lower in the CD/RALA, RALA, and CD groups than in the C group (Figure 4).

### 3.3. O_2_^−^ Radical Scavenging

**Figure 5:**
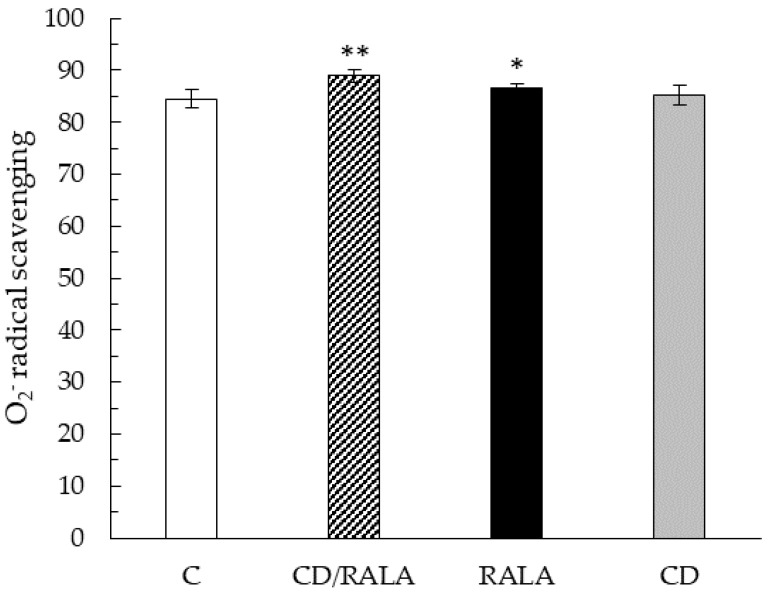
O_2_^−^ radical scavenging activity (%) of the kidney. Values are expressed as the mean ± SD for six mice in each group. C, control group; CD/RALA, R-α-lipoic acid γ-cyclodextrin complex group; RALA, R-α-lipoic acid group; CD, γ-cyclodextrin group. ** *p* < 0.01 vs. C. * *p* < 0.05 vs. C.

The O_2_^−^ scavenging activity of the kidney was significantly higher in the CD/RALA and RALA groups than in the C group, and the highest value was observed in the CD/RALA group (Figure 5). The O_2_^−^ radical scavenging activity of the liver was significantly lower in the CD group than in the C group. No differences were observed in the activity of other organs and muscles (Table 3).

### 3.4. Trace Element Analysis

**Figure 6:**
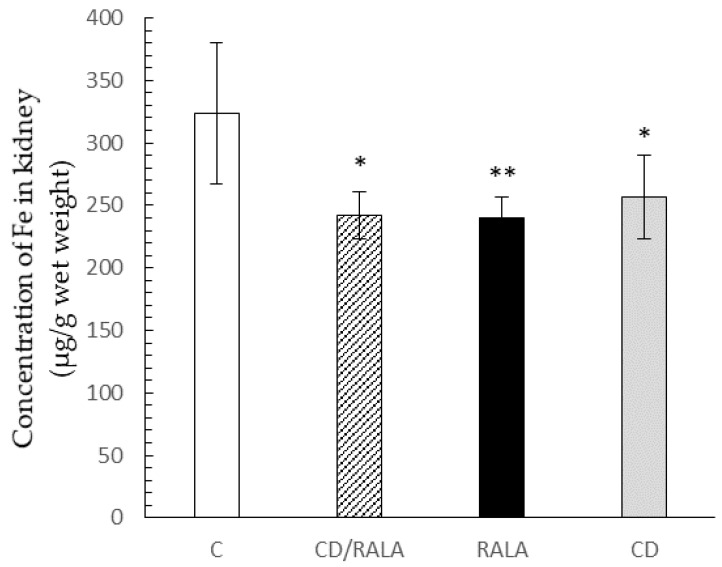
Concentration of Fe in the kidney. Values are expressed as the mean ± SD for six mice in each group. C, control group; CD/RALA, R-α-lipoic acid γ-cyclodextrin complex group; RALA, R-α-lipoic acid group; CD, γ-cyclodextrin group. ** *p* < 0.01 vs. C, * *p* < 0.05 vs. C.

The concentration of Fe in the kidney was significantly lower in the CD/RALA, RALA, and CD groups than in the C group (Figure 6).

### 3.5. Other Experiments

Swimming exercise time to exhaustion for the CD/RALA, RALA, and CD groups were longer than for the C group (Table 4). The number of mice that swam up to the upper limit of 60 min was one in the C group, five in the CD/RALA group, three in the RALA group, and four in the CD group.

The difference in blood lactate levels before and after swimming exercise tended to be highest in the CD/RALA group (Table 4).

Blood CPK activity tended to be lower in the CD/RALA, RALA, and CD groups compared to the C group (Table 4).

### 3.6. Histopathological and Immunohistochemical Examination of the Kidney

**Figure 7:**
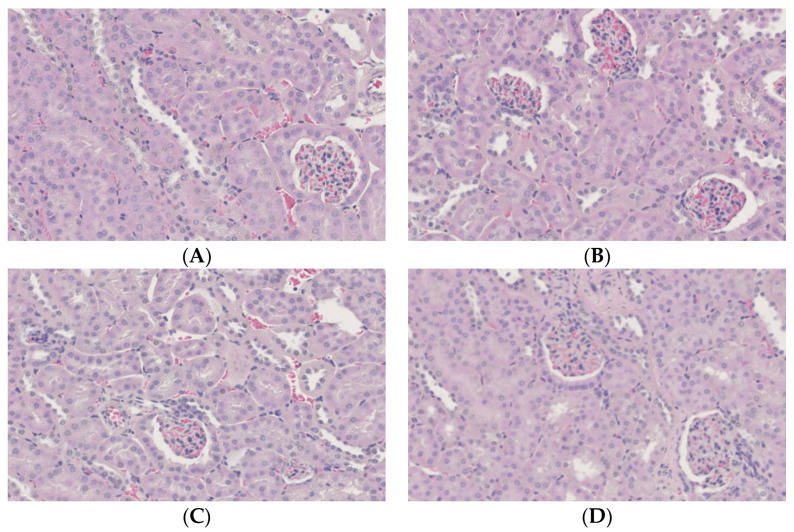
Histopathological changes in the kidneys of mice. (**A**) C, control group; (**B**) CD/RALA, R-α-lipoic acid γ-cyclodextrin complex group; (**C**) RALA, R-α-lipoic acid group; (**D**) CD, γ-cyclodextrin group.

No histopathological changes were detected in any groups (Figure 7). Immunostaining of kidney sections with antibodies to carbonylated protein, a marker of oxidative stress, showed no difference between the groups.

## 4. Discussion

We investigated whether RALA complexed with γCD is effective in enhancing exercise performance, and we obtained a great deal of characteristic data in this experiment.

Blood NH_3_ concentration was significantly lower in the CD/RALA, RALA, and CD groups than in the C group (Figure 4). The direct source of NH_3_ is thought to be deamination of AMP, which accumulates with exercise and has been reported to be a marker of fatigue [34,35,36]. Therefore, decreased NH_3_ generation can reduce fatigue, which may lead to improved endurance. Although there was no significant difference in the marginal swimming exercise time, there was a tendency for each intervention group to extend the time compared to group C (Table 4). In this experiment, the upper time limit was set at one hour so as not to overstress the mice; five mice in the CD/RALA group swam up to the upper time limit of one hour, which was more than those in the C, RALA, and CD groups. If there was no limit to the exercise time, the CD/RALA group may have extended the time further than the present results. These results suggest that high ammonia concentration may shorten the marginal exercise time. This means that each intervention group may have reduced fatigue and improved endurance by suppressing the increase in ammonia concentration. Given that CD is a carbohydrate, the concentration of NH_3_ in the CD/RALA and RALA groups was suppressed. The CD group was in a high-energy state due to carbohydrate intake because CD is a carbohydrate, which may have reduced fatigue.

The O_2_^−^ scavenging activity of the kidney was significantly higher in the CD/RALA and RALA groups than in the C group (Figure 5). The blood CPK activity was not significantly different, but tended to be highest in the C group. It has also been reported that exercise increases ROS and total CPK activity, suggesting that muscle damage is a key factor related to increased ROS production during exercise [37,38,39,40,41]. In the present study, the results of CPK activity in blood may also support the results of O_2_^−^ scavenging activity in the kidney (Table 4).

Meo et al. reported that intensive exercise generates ROS in the body [42]. The main process that generates ROS is thought to be electron transport associated with the mitochondrial respiratory chain. It is widely believed that during exercise, the rate of ROS production increases due to the increased flow of electrons through the electron transport chain of mitochondria. The mitochondrial respiratory chain is a potential source of ROS in the kidney, which is partially ischemic due to reduced blood supply during exercise. It is believed that sufficient oxygen can interact with the reduced respiratory chain to inhibit ROS generation. ALA intake has been reported to protect mitochondria, as indicated above [43]. The O_2_^−^ scavenging activity of CD/RALA was more than that of RALA in the present study, suggesting that the antioxidant and mitochondrial protective effects of RALA were enhanced by CD inclusion. In fact, CD/RALA inhibited excessive oxidative stress in the kidneys caused by swimming exercise.

It has been reported that when Fe is present in excess amounts in the body, hydroxyl radicals (-OH), which are highly reactive among ROS, are produced via the Fenton reaction [43]. Trace element analysis of the kidney showed that Fe concentration was significantly lower in all groups than in the control group (Figure 6). This result shows a similar trend to the O_2_^−^ scavenging activity of the kidney, suggesting that there is a relationship between oxidative stress and Fe concentration in the kidneys. 

These results suggest that CD/RALA has a positive effect on exercise performance. In addition, it was confirmed that the antioxidant potential of RALA was enhanced by CD. The results suggested that it suppressed the increased NH_3_ concentration caused by exercise. These effects may have contributed to the tendency to prolong the exercise time to the limit in the CD/RALA group. Although the relationship between exercise, oxidative stress, and muscle fatigue has been demonstrated, the underlying mechanism remains unclear. There are few reports on the burden of exercise on the kidneys, although in the present experiment, the burden of exercise on the kidneys was clearly observed. It has been reported that alpha lipoic acid has anti-inflammatory effects as well as antioxidant effects [44]. It is possible that these functions may be involved in this experiment as well. Exploring these related mechanisms will be the subject of future research. In the present study, a lipoic acid dose of 50 mg/kg BW was administered to mice, and we obtained the above results. However, as for human intake, the amount of alpha lipoic acid contained in commercial foods is 50-200 mg per day, and the dosage in this case is quite large. Therefore, when applying this product to humans in the future, we believe that the form of intake and the amount of intake need to be considered [45].

## 5. Conclusions

In male C3H/HeSlc mice, there was suppression of the increased blood ammonia level, suppression of the blood CPK activity, and an increase in the antioxidant capacity of the kidney in the CD/RALA-treated group compared to the non-CD/RALA-treated groups. The improvement in endurance in the CD/RALA group may be due to these factors.

## Figures and Tables

**Table 1:** Experimental groups and dose of administered substance.

Groups	Compounds	Solvent (mL/g BW)	Dose (mg/kg BW)
C	Distilled Water	0.01	-
CD/RALA	RALA-γCD	0.01	50
RALA	RALA	0.01	50
CD	γCD	0.01	50

**Table 2:** General characteristics of animals.

Organs	C	CD/RALA	RALA	CD
Body weight (g)	22.5 ± 1.8	23.3 ± 1.4	22.1 ± 1.0	22.8 ± 1.0
Food intake (g/day)	3.0 ± 0.4	3.0 ± 0.5	2.8 ± 0.5	2.9 ± 0.3
Liver (mg/kg BW)	47.6 ± 1.9	47.4 ± 4.8	44.2 ± 1.5	45.0 ± 2.3
Kidney (mg/kg BW)	12.7 ± 0.8	13.0 ± 0.9	13.2 ± 0.7	12.5 ± 0.6
Spleen (mg/kg BW)	2.6 ± 0.2	2.6 ± 0.5	2.5 ± 0.2	2.5 ± 0.2
Lung (mg/kg BW)	6.8 ± 0.3	7.4 ± 1.6	7.4 ± 0.6	7.6 ± 0.4
Heart (mg/kg BW)	4.8 ± 0.3	4.9 ± 0.4	4.8 ± 0.4	4.6 ± 0.3

Data are expressed as the mean ± SD.

**Table 3:** O_2_^−^ radical scavenging activity (%) of each organ.

Organs	C	CD/RALA	RALA	CD
Liver	88 ± 1	88 ± 2	88 ± 1	84 ± 3 *
Kidney	84 ± 2	89 ± 1 **	87 ± 1 *	85 ± 2
Spleen	57 ± 9	51 ± 14	51 ± 11	62 ± 5
Lung	37 ± 8	38 ± 6	30 ± 5	24 ± 10
Quadriceps	44 ± 8	41 ± 10	34 ± 11	37 ± 4
Gastrocnemius	23 ± 9	20 ± 11	20 ± 14	8 ± 14

Values are expressed as the mean ± SD for six mice in each group. *^,^ ** Significant difference from the control group (*p* < 0.05, *p* < 0.01). C, control group; CD/RALA, R-α lipoic acid γ-cyclodextrin complex group; RALA, R-α lipoic acid group; CD, γ-cyclodextrin group.

**Table 4:** Other experiments.

Experimental item	C	CD/RALA	RALA	CD
Swimming exercise time to exhaustion (minutes)	49.9 ± 6.1	57.0 ± 6.7	56.7 ± 4.6	52.1 ± 12.3
Blood lactate level defference before and after training (nmol/L)	1.5 ± 1.0	2.5 ± 1.3	1.3 ± 1.1	1.7 ± 1.5
Blood CPK activity (U/L)	101 ± 29	80 ± 22	69 ± 25	88 ± 22

Values are expressed as the mean ± SD for six mice in each group. C, control group; CD/RALA, R-α lipoic acid γ-cyclodextrin complex group; RALA, R-α lipoic acid group; CD, γ-cyclodextrin group.

## Data Availability

Data sharing is not applicable to this article.
